# Expression of HLA class I is associated with immune cell infiltration and patient outcome in breast cancer

**DOI:** 10.1038/s41598-022-24890-3

**Published:** 2022-11-27

**Authors:** Song-Hee Han, Milim Kim, Yul Ri Chung, Ji Won Woo, Hye Yeon Choi, So Yeon Park

**Affiliations:** 1 Department of Pathology, Seoul National University College of Medicine, Seoul, Republic of Korea; 2 Department of Pathology, Dong-A University College of Medicine, Busan, Republic of Korea; 3 Department of Pathology, Seoul National University Bundang Hospital, 82, Gumi-Ro 173 Beon-Gil, Bundang-Gu, Seongnam, Gyeonggi 13620 Republic of Korea; 4 Pathology Center, Seegene Medical Foundation, Seoul, Republic of Korea

**Keywords:** Cancer, Immunology

## Abstract

Downregulation of human leukocyte antigen (HLA) class I is one mechanism of escaping anti-tumor immunity by tumor cells. This study was conducted to compare HLA class I expression in ductal carcinoma in situ (DCIS) and invasive breast carcinoma (IBC) and to evaluate its association with immune cell infiltration of the tumors and clinical outcome of the patients. A total of 830 cases comprising 288 DCIS and 542 IBC were included in this study. Immunohistochemistry for HLA class I expression was performed using HLA-ABC in tissue microarrays and was analyzed in relation to clinicopathologic characteristics of tumors and infiltration of CD4+, CD8+, and FOXP3+ tumor-infiltrating lymphocyte (TIL) subsets and PD-L1+ immune cells. As a whole, there was no difference in HLA class I expression between DCIS and IBC when dichotomized into high or low expression. However, in the HR-negative group, a high level of HLA class I expression was more frequent in IBC than DCIS. On the contrary, in the HR-positive group, a complete loss of HLA class I expression was more frequently observed in IBC than DCIS. High HLA class I expression level was generally associated with aggressive clinicopathologic features of IBC and was associated with high CD4+, CD8+, and FOXP3+ TIL and PD-L1+ immune cell infiltration in both DCIS and IBC. In survival analyses, HLA class I expression was not associated with clinical outcome in DCIS and IBC as a whole; however, low HLA class I expression was associated with poor clinical outcome in HR-negative IBC, especially in triple-negative subtype. In conclusion, this study showed that HLA class I expression increased in association with increased immune cell infiltration during in situ to invasive transition of HR-negative breast cancer, and HLA class I down-regulation had a prognostic value in HR-negative breast cancer.

## Introduction

Tumor immune microenvironment is regarded as an important element in cancer development, progression, and control^1^. Immune reactions mediated by CD8+ and CD4+ Th1 lymphocytes play a crucial role in anti-tumor immunity by eliciting specific immune responses to tumors. While regulatory T cells (Tregs) have an essential role in maintaining immune tolerance, Tregs also have a strong immunosuppressive function by inhibiting activation and differentiation of CD4+ and CD8+ T lymphocytes^2^. In addition to these immune cells, programmed cell death-ligand 1 (PD-L1) is an important immune checkpoint molecule expressed on both cancer cells and immune cells^3^ that serves as a brake in an immune reaction by interfering with activation of T lymphocytes. Thus, the composition and quantity of these immune cell subsets affect the balance between their pro- and anti-tumor effects, which in turn, modulate the course of cancer^4,5^.

Ductal carcinoma in situ (DCIS), which is characterized by proliferation of tumor cells within the ductal-lobular system, is a non-obligate precursor of invasive breast carcinoma (IBC)^6^. Although previously uncommon, it now comprises up to 25% of all breast cancers, and up to 40% can progress to IBC^7^. However, it remains ambiguous how DCIS progresses to IBC and what factors determine the transition to invasive carcinoma although it is thought to be a complex process driven by tumor cells and tumor microenvironment including myoepithelial cells, stromal fibroblasts, and immune infiltrates^8^.

DCIS commonly shows a significant amount of immune cell infiltration which is frequently found in the periductal stroma^9^. Dense tumor-infiltrating lymphocyte (TIL) infiltration is more prevalent in high-grade DCIS, especially in the hormone receptor (HR)-negative subgroup^10,11^. Our previous study demonstrated the different composition of immune cell subsets between DCIS and invasive carcinoma depending on the HR status^11^. Interestingly, more immune cell infiltration has been associated with tumor recurrence^12,13^. These findings support a hypothesis that immunological composition and immune-related dynamic changes are strongly related to subsequent tumor progression in DCIS.

Evasion of immune surveillance is essential in tumor development^14^, and down-regulation of human leukocyte antigen (HLA) class I molecule is a well-established mechanism of immune evasion in cancer^15^. HLA class I is expressed on the cell membrane in most cells and triggers important activities of immune surveillance, presenting cellular peptides to cytotoxic T lymphocytes^16^. Thus, deficient HLA class I expression can lead to inactivation of T-cell-based immune surveillance resulting in tumor development and progression^16^. Various mechanisms underlying loss or down-regulation of HLA class I have been reported: promoter methylation, mutations in the HLA class I heavy chains, ꞵ2-microglobulin or antigen presentation machinery components, loss of heterozygosity of HLA gene loci, and transcriptional regulation^17^. Downregulation of HLA class I has been reported in 40–70% of invasive cancer^18–20^, and it has often shown correlations with worse prognoses^17–19,21–24^.

To our knowledge, however, there exists no previous study comparing HLA class I expression in in situ and invasive carcinoma of the breast from the standpoint of balance in immune response. Tumor cells in DCIS, being confined to the ductal-lobular system, are physically separated from the stromal immune cells and become directly intermingled with these immune cells during invasive progression. We supposed that invasive tumor cells, which are inevitably recognized by the immune cells in the stroma, require an immune escape for survival via mechanisms such as alteration of HLA class I expression, and that this would lead to a difference in HLA class I expression between DCIS and IBC. This study was aimed at comparing HLA class I expression in DCIS and IBC and evaluating the associations between HLA class I expression, immune cell subset infiltration, and clinical outcome of the patients with DCIS and IBC.

## Results

### Comparison of HLA class I expression between DCIS and IBC

HLA class I expression was mostly detected in the tumor cell membrane showing a complete membranous staining pattern; however, occasional incomplete membranous staining or cytoplasmic staining was observed. Only complete or incomplete membranous staining was evaluated in this study.

**Figure 1:**
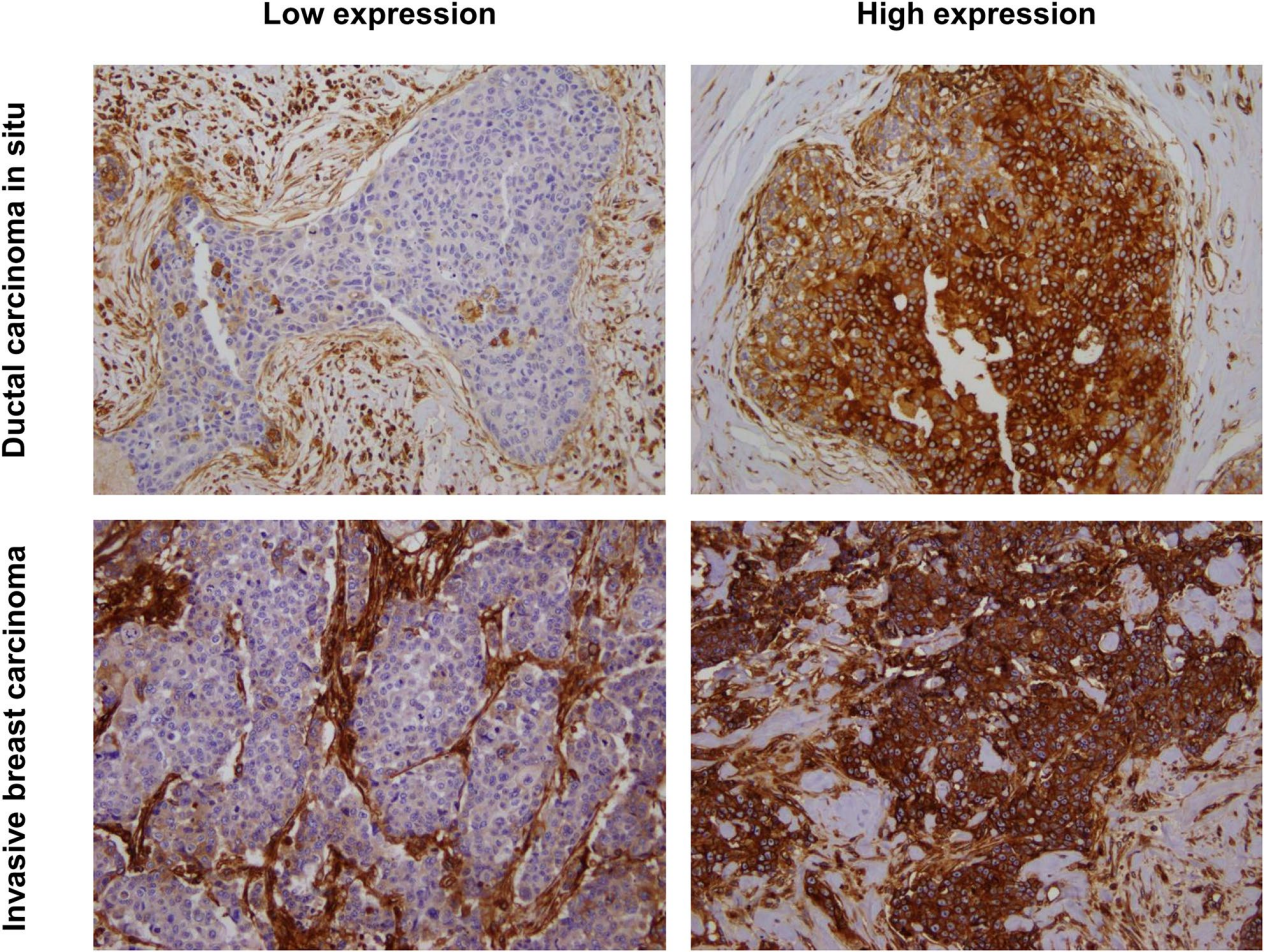
Representative images of immunostaining with human leukocyte antigen (HLA) class I in ductal carcinoma in situ (DCIS) and invasive breast carcinoma (IBC). In the low level expression group, tumor cells exhibit a complete loss of membranous expression or weak, focal membranous expression of HLA class I compared to the stromal cells that show strong expression. Conversely, tumor cells in the high level expression group show mostly diffuse and strong, complete membranous expression in both DCIS and IBC.

First, HLA class I expression was compared between DCIS and IBC using 830 breast cancer samples comprising 288 cases of DCIS and 542 cases of IBC (Table 1; Fig. 1). As a whole, there was no difference in HLA class I expression in DCIS and invasive carcinoma when it was dichotomized into high and low expression. Low HLA class I expression, defined as strong positivity in < 50% of the tumor cells or weak positivity in ≥ 50% of tumor cells, was found in 145 cases (50.3%) of DCIS and 269 cases (49.6%) of IBC. In the HR-positive group, low HLA class I expression tended to be more frequent in IBC compared to DCIS (53.4% vs. 46. 9%; *p* = 0.122). On the contrary, in the HR-negative group, low HLA class I expression was more frequent in DCIS than in IBC (62.9% vs. 40.5%); high HLA class I expression was more frequently found in IBC than in DCIS (59.5% vs. 37.1%; *p* = 0.003).

**Table 1:** Comparison of HLA class I expression in DCIS and IBC.

Group	HLA class I expression	DCIS (n = 288)	IBC (n = 542)	*p* value
Total	Low	145 (50.3%)	269 (49.6%)	0.844
	High	143 (49.7%)	273 (50.4%)	
	Complete loss	27 (9.4%)	92 (17.0%)	0.003
HR-positive	Low	106 (46.9%)	205 (53.4%)	0.122
	High	120 (53.1%)	179 (46.6%)	
	Complete loss	22 (9.7%)	68 (17.7%)	0.007
HR-negative	Low	39 (62.9%)	64 (40.5%)	0.003
	High	23 (37.1%)	94 (59.5%)	
	Complete loss	5 (8.1%)	24 (15.2%)	0.160

*p* values are calculated by Chi-square test.

*DCIS* ductal carcinoma in situ, *IBC* invasive breast carcinoma, *HLA* human leukocyte antigen.

A complete loss of HLA class I expression was more frequently observed in IBC than in DCIS (17.0% vs. 9.4%; *p* = 0.003). In subgroup analysis by HR status, the difference was also apparent in the HR-positive group (17.7% vs. 9.7%; *p* = 0.007) but with borderline significance in the HR-negative subgroup (15.2% vs. 8.1%; *p* = 0.160).

Next, we compared HLA class I expression in DCIS and invasive components within same tumors using 90 cases of IBC with a DCIS component (Table 2). HLA class I expression level was not statistically different between DCIS and invasive components of the same tumor in the whole group, and HR-positive and HR-negative subgroups (*p* = 0.238, *p* = 0.302 and *p* = 1.000, respectively). A complete loss of HLA class I expression tended to be more frequent in the invasive component than in the DCIS component with borderline significance in the whole group (*p* = 0.092).

**Table 2:** Comparison of HLA class I expression in the DCIS and invasive components within same tumors.

Hormone receptor status	Low in invasive component/High in DCIS component	High in invasive component/Low in DCIS component	High in invasive component/High in DCIS component	Low in invasive component/Low in DCIS component	*p* value
Total (n = 90)	12	6	40	32	0.238
HR-positive (n = 67)	10	5	27	25	0.302
HR-negative (n = 23)	2	1	13	7	1.000

Hormone receptor status	Complete loss in invasive component/Any expression in DCIS component	Any expression in invasive component/Complete loss in DCIS component	Any expression in invasive component/Any expression in DCIS component	Complete loss in invasive component/Complete loss in DCIS component	*p* value
Total (n = 90)	10	3	76	1	0.092
HR-positive (n = 67)	7	2	57	1	0.180
HR-negative (n = 23)	3	1	19	0	0.625

*p* values were calculated by McNemar test.

*HLA* human leukocyte antigen, *DCIS* ductal carcinoma in situ.

The difference in HLA class I expression between DCIS and DCIS associated with invasive carcinoma (DCIS-INV) was also examined (Supplementary Table S1). HLA class I expression level was not significantly different between DCIS and DCIS-INV in the whole group and in the HR-positive group (*p* = 0.809 and *p* = 0.443, respectively). However, in the HR-negative group, a high level of HLA class I expression tended to be more frequently found in DCIS-INV than in DCIS (60.9% vs. 37.1%; *p* = 0.050). The frequency of HLA class I loss was not different between DCIS and DCIS-INV in all groups.

### Correlation of HLA class I expression with clinicopathologic features of tumor

In DCIS, HLA class I expression level was associated with only ER status: low HLA class I expression was associated with ER-negative tumors (*p* = 0.020; Table 3). Low HLA class I expression level was more common in HER2-positive and triple-negative subtypes compared to luminal A and luminal B subtypes.

**Table 3:** Relationship between HLA class I expression level and clinicopathologic features of DCIS.

Clinicopathologic feature	HLA class I expression		*p* value
	Low (n = 145)	High (n = 143)	
**Extent (cm)**			0.341
< 3.0	72 (49.7)	63 (44.1)	
≥ 3.0	73 (50.3)	80 (55.9)	
**Nuclear grade**			0.474
Low to intermediate	74 (51.0)	79 (55.2)	
High	71 (49.0)	64 (44.8)	
**Comedo-type necrosis**			0.674
Absent	96 (66.2)	98 (68.5)	
Present	49 (33.8)	45 (31.5)	
**Microinvasion**			0.316
Absent	110 (75.9)	101 (70.6)	
Present	35 (24.1)	42 (29.4)	
**Estrogen receptor**			0.020
Negative	41 (28.3)	24 (16.8)	
Positive	104 (71.7)	119 (83.2)	
**Progesterone receptor**			0.175
Negative	47 (32.4)	36 (25.2)	
Positive	98 (67.6)	107 (74.8)	
**HER2 status**			0.629
Negative	113 (77.9)	108 (75.5)	
Positive	32 (22.1)	35 (24.5)	
**Ki-67 index**			0.514
Low (< 10%)	91 (62.8)	95 (66.4)	
High (≥ 10%)	54 (37.2)	48 (33.6)	
**P53 overexpression**			0.509
Absent	120 (82.8)	114 (79.7)	
Present	25 (17.2)	29 (20.3)	
**Subtype**			0.005
Luminal A	93 (64.1)	88 (61.5)	
Luminal B	13 (9.0)	32 (22.4)	
HER2+	23 (15.9)	16 (11.2)	
Triple negative	16 (11.0)	7 (4.9)	

*p* values are calculated by Chi-square test.

*HLA* human leukocyte antigen, *DCIS* ductal carcinoma in situ.

However, the relationship between HLA class I expression level and clinicopathologic features of IBCs revealed different patterns (Table 4). Interestingly, high HLA class I expression was associated with aggressive clinicopathologic features including high histologic grade (*p* < 0.001), ER negativity (*p* = 0.002), positive HER2 status (*p* = 0.032), high Ki-67 labeling index (*p* < 0.001) and p53 overexpression (*p* < 0.001). Accordingly, a high level of HLA class I expression was predominant in HER2+ and triple-negative subtypes of IBCs with a significant difference between DCIS and IBC in triple-negative subtype (*p* = 0.004).

**Table 4:** Relationship between HLA class I expression level and clinicopathologic features of IBC.

Clinicopathologic features	HLA class I expression		*p* value
	Low	High	
**T stage**			0.859
T1	129 (48.0)	133 (48.7)	
T2-T4	140 (52.0)	140 (51.3)	
**N stage**			0.087
N0	143 (53.2)	165 (60.4)	
N1-N3	126 (46.8)	108 (39.6)	
**Histologic grade**			< 0.001
I-II	160 (59.5)	105 (38.5)	
III	109 (40.5)	168 (61.5)	
**Lymphovascular invasion**			0.586
Absent	156 (58.0)	152 (55.7)	
Present	113 (42.0)	121 (44.3)	
**Estrogen receptor**			0.002
Negative	66 (24.5)	101 (37.0)	
Positive	203 (75.5)	172 (63.0)	
**Progesterone receptor**			0.238
Negative	99 (36.8)	114 (41.8)	
Positive	170 (63.2)	159 (58.2)	
**HER2 status**			0.032
Negative	217 (80.7)	199 (72.9)	
Positive	52 (19.3)	74 (27.1)	
**Ki-67 index**			
Low (< 20%)	178 (66.2)	127 (46.5)	< 0.001
High (≥ 20%)	91 (33.8)	146 (53.5)	
**P53 overexpression**			< 0.001
Absent	221 (82.2)	180 (65.9)	
Present	48 (17.8)	93 (34.1)	
**Subtype**			< 0.001
Luminal A	144 (53.5)	88 (32.2)	
Luminal B	61 (22.7)	91 (33.3)	
HER2+	30 (11.2)	34 (12.5)	
Triple negative	34 (12.6)	60 (22.0)	

*p* values are calculated by Chi-square test.

*HLA* human leukocyte antigen, *IBC* invasive breast carcinoma.

A complete loss of HLA class I expression did not show correlations with clinicopathologic features of tumors except for absence of microinvasion (*p* = 0.005) in DCIS (Supplementary Table S2) and low Ki-67 proliferation index (*p* = 0.033) as well as absence of p53 overexpression (*p* = 0.020) in IBCs (Supplementary Table S3). Thus, further analyses were focused on HLA class I expression levels thereafter.

### Comparison of immune cell subsets between DCIS and invasive carcinoma according to the expression level of HLA class I

In the next step, we compared immune cell subset infiltration according to HLA class I expression levels using 288 cases of DCIS and 339 cases of IBC to check whether immune cell infiltration is associated with HLA class I expression in tumor cells (Table 5). All TIL subset infiltration and the presence of PD-L1+ immune cells were significantly higher in IBC compared to DCIS as previously have been shown^11^. As a whole, in both DCIS and IBC, tumors with high HLA class I expression contained significantly more CD4+, CD8+, and FOXP3+ TILs and PD-L1+ immune cells than those with low HLA class I expression (all *p* < 0.05). The difference was also evident in HR-positive subgroup except for CD4+ TIL infiltration in IBC. In HR-negative group, high infiltration of CD4+, CD8+ and FOXP3+ TILs and PD-L1+ immune cells were also associated with high HLA class I expression in both DCIS and IBC, albeit with borderline significance for CD4+ and CD8+ TIL infiltration in DCIS and CD4+ TIL infiltration in IBC.

**Table 5:** Comparison of immune cell subset infiltration in relation to HLA class I expression.

Immune cell subset	DCIS		*p* value	IBC		*p* value
	Low HLA class I expression	High HLA class I expression		Low HLA class I expression	High HLA class I expression	
**Whole group**						
CD4+ TIL	17.7 (4.0–43.0)	40.3 (10.6–94.3)	< 0.001	73.0 (36.0–152.0)	104.0 (45.0–178.0)	0.032
CD8+ TIL	11.7 (5.0–22.0)	15.0 (6.2–36.0)	0.007	60.0 (32.0–128.0)	112.0 (56.5–239.5)	< 0.001
FOXP3+ TIL	0.0 (0.0–1.0)	0.0 (0.0–4.0)	0.003	5.0 (2.0–12.0)	13.0 (5.5–24.0)	< 0.001
PD-L1+ IC	17/144 (11.8)	37/137 (27.0)	0.001	46/151 (30.5)	93/168 (55.4)	< 0.001
**HR+ subgroup**						
CD4+ TIL	10.0 (2.0–31.2)	31.0 (8.7–76.0)	< 0.001	71.0 (38.5–147.5)	86.5 (43.8–156.8)	0.233
CD8+ TIL	9.0 (4.0–17.3)	13.3 (5.7–30.7)	0.005	56.0 (31.5–116.0)	84.0 (43.5–187.8)	0.002
FOXP3+ TIL	0.0 (0.0–0.0)	0.0 (0.0–2.0)	< 0.001	5.0 (2.0–10.5)	9.0 (4.0–20.0)	< 0.001
PD-L1+ IC	9/105 (8.6)	23/115 (20.0)	0.016	19/37 (51.4)	52/59 (88.1)	< 0.001
**HR− subgroup**						
CD4+ TIL	43.0 (22.3–77.0)	92.3 (34.8–125.3)	0.094	820. (11.5–154.0)	127.0 (48.0–218.0)	0.068
CD8+ TIL	15.7 (9.0–36.3)	44.2 (10.2–74.1)	0.065	110.5 (37.0–190.8)	192.0 (108.0–320.0)	0.003
FOXP3+ TIL	2.0 (0.0–5.0)	8.0 (0.8–14.3)	0.025	8.0 (4.0–18.0)	18.0 (11.0–31.0)	< 0.001
PD-L1+ IC	8/39 (20.5)	14/22 (63.6)	0.001	27/114 (23.7)	41/109 (37.6)	0.029

*p* values are calculated by Mann–Whitney U test or Chi-square test. Data are presented as median (interquartile range) for TIL and frequency (%) for PD-L1+ IC.

*HLA* human leukocyte antigen, *DCIS* ductal carcinoma in situ, *IBC* invasive breast carcinoma.

### Prognostic significance of HLA class I expression level in breast carcinoma

**Figure 2:**
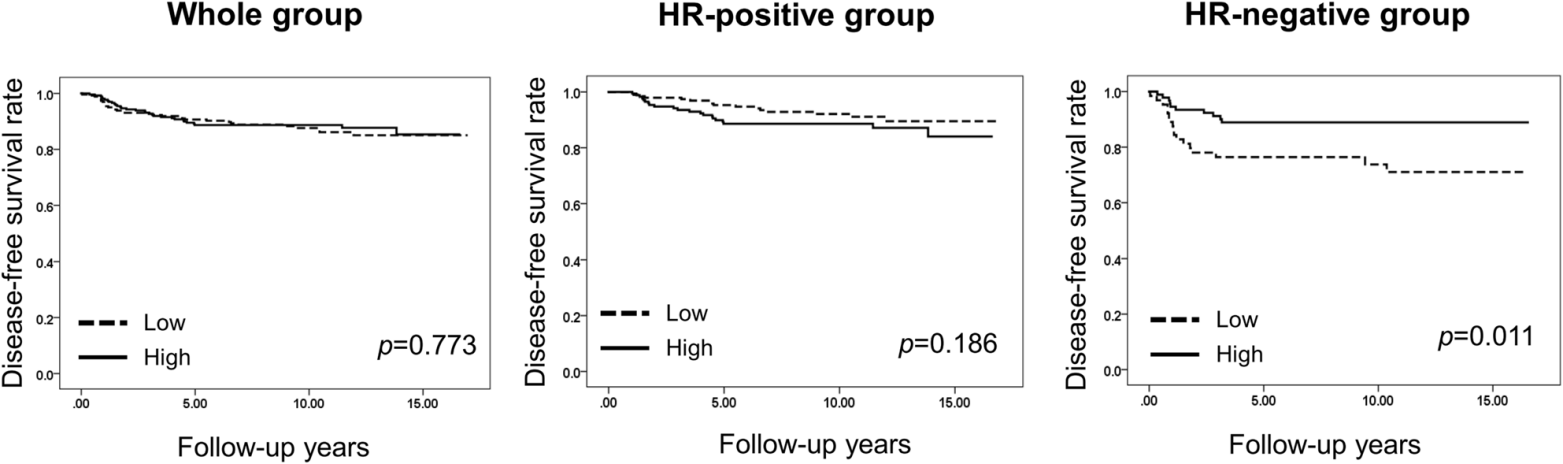
Kaplan–Meier survival curves according to the expression level of human leukocyte antigen (HLA) class I in patients with invasive breast carcinoma. Low HLA class I expression is associated with shorter disease-free survival of patients in the hormone receptor (HR)-negative subgroup. There is no survival difference in the whole group and in the HR-positive subgroup.

In DCIS, expression level of HLA class I did not show a statistically significant association with recurrence-free survival of patients with DCIS, irrespective of HR status (*p* = 0.415, whole group; *p* = 0.524, HR-positive group; *p* = 0.929, HR-negative group; Supplementary Fig. 1). In IBC, HLA class I expression level was not associated with disease-free survival of the patients as a whole. However, in subgroup analysis, low expression of HLA class I was associated with significantly worse disease-free survival in the HR-negative subgroup (*p* = 0.011), but not in the HR-positive subgroup (*p* = 0.186; Fig. 2). Furthermore, in multivariate analysis, expression level of HLA class I was revealed as an independent prognostic factor (Hazard ratio, 2.238; 95% CI, 1.021–4.907; *p* = 0.044; Table 6).

**Table 6:** Univariate and multivariate analyses of disease-free survival in the HR-negative and triple-negative IBC.

Variable	Category	Univariate analysis		Multivariate analysis	
		Hazard ratio (95% CI)	*p* value	Hazard ratio (95% CI)	*p* value
**HR-negative IBC**					
Age	< 50 years vs. ≥ 50 years	1.248 (0.586–2.655)	0.566		
T stage	T2-4 vs. T1	1.714 (0.750–3.916)	0.201		
N stage	N1-N3 vs. N0	2.253 (1.059–4.793)	0.035	1.040 (0.445–2.431)	0.927
Histologic grade	III vs. I & II	1.320 (0.397–4.385)	0.650		
LVI	Present vs. Absent	5.265 (2.225–12.458)	< 0.001	4.814 (2.026–11.442)	< 0.001
HER2	Negative vs. Positive	1.384 (0.622–3.082)	0.426		
Ki-67 index	High vs. Low	1.107 (0.383–3.201)	0.851		
HLA class I	Low vs. High	2.646 (1.211–5.780)	0.015	2.238 (1.021–4.907)	0.044
**Triple-negative IBC**					
Age	< 50 years vs. ≥ 50 years	0.968 (0.382–2.454)	0.945		
T stage	T2-4 vs. T1	3.034 (0.878–10.485)	0.079	1.585 (0.432–5.818)	0.488
N stage	N1-N3 vs. N0	2.744 (1.089–6.918)	0.032	0.949 (0.315–2.857)	0.926
Histologic grade	III vs. I & II	0.959 (0.220–4.171)	0.955		
LVI	Present vs. Absent	6.583 (2.344–18.493)	< 0.001	6.272 (2.229–17.649)	0.001
Ki-67 index	High vs. Low	0.952 (0.219–4.141)	0.948		
HLA class I	Low vs. High	2.901 (1.124–7.486)	0.028	2.654 (1.027–6.858)	0.044

*HR* hormone receptor; *IBC* invasive breast carcinoma, *CI* confidence interval, *LVI* lymphovascular invasion.

**Figure 3:**
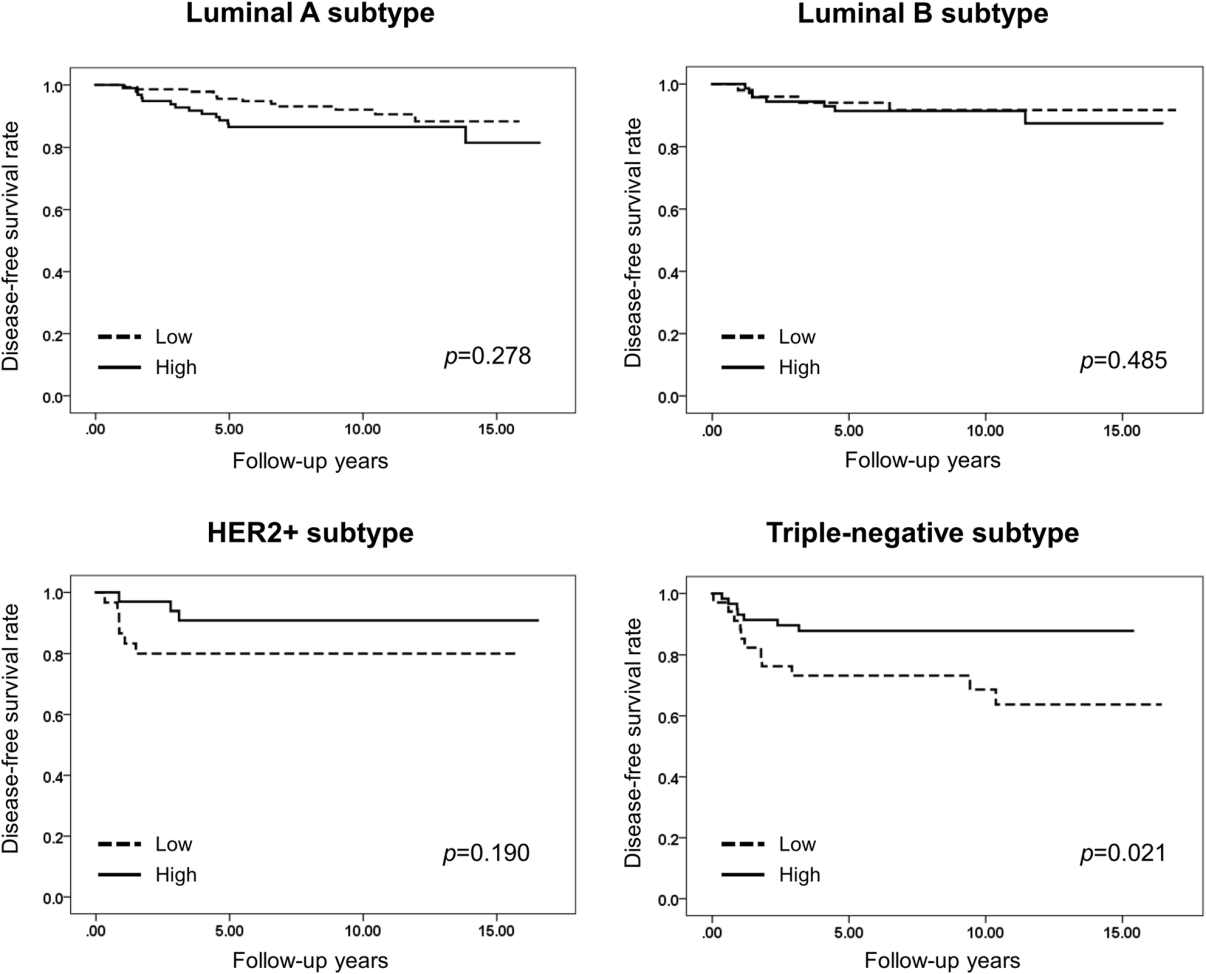
Kaplan–Meier survival curves stratified by expression level of human leukocyte antigen (HLA) class I in different subtypes of invasive breast carcinoma. Low HLA class I expression is associated with decreased disease-free survival in triple-negative subtype, but not in luminal A, luminal B, and HER+ subtypes.

We also performed survival analyses according to subtype of breast cancer including luminal A, luminal B, HER2+ and triple-negative. Low expression of HLA class I was associated with decreased disease-free survival of the patients with triple-negative breast cancer (TNBC) (*p* = 0.021; Fig. 3). Furthermore, in multivariate analysis, low HLA class I expression level was found to be an independent adverse prognostic factor for disease-free survival in TNBC (Hazard ratio, 2.654; 95% CI, 1.027–6.858; *p* = 0.044; Table 6).

## Discussion

As HLA class I down-regulation is a well-known mechanism of immune evasion in cancer and is associated with tumor progression^15^, we had expected that IBC would show decreased HLA class I expression compared to DCIS. While a complete loss of HLA class I was more frequent in IBC than in DCIS, its expression level was not different between IBC and DCIS. Interestingly, in the HR-negative subgroup, high HLA class I expression was more frequent in IBC than in DCIS, and it also tended to be higher in DCIS-INV than in DCIS. Although HLA class I molecules are known to be expressed at the basal state in almost all nucleated cells, this expression can be induced or repressed through various mechanisms including interferon signaling and transcription factors of nuclear factor-κB (NF-κB) and NLRC5, etc. by the cell itself or its surrounding immune cells^25^. Thus, we suppose that increased HLA class I expression in IBC compared to DCIS in HR-negative tumors would be related to the degree of immune cell infiltration and induction of HLA class I by the immune cells as IBC generally shows higher immune cell infiltration than DCIS, which is a finding more prominent in HR-negative tumors.

In breast cancer, expression of HLA class I has been linked to aggressive features of tumor. Shin et al.^17^ observed that HLA class I expression correlated with lymph node metastasis and high histopathological grade. Park et al.^23^ reported a positive correlation between HLA class I expression and high histologic and nuclear grades. Lee et al.^26^ demonstrated that HLA class I expression was negatively correlated with ER expression. Similarly, we observed that high HLA class I expression was associated with aggressive features of tumor such as high histologic grade, ER negativity, positive HER2 status, high Ki-67 proliferation index, and p53 overexpression. Moreover, in this study, high HLA class I expression was associated with high TIL subset and PD-L1+ immune cell infiltration. These associations could be explained by the study by Takahashi and colleagues who demonstrated the counterbalance among aggressive tumor features, accumulation of mutation, and strong immunogenicity in breast cancer^27^. They also showed that aggressive tumor behaviors were associated with a high mutational burden, which was more frequent in the ER-negative subgroup and TNBC, and to increased neo-antigen loads^27^. Several studies demonstrated that neo-antigens attract effective immune cells in their microenvironments such as CD4+ T cells or M1 macrophages^27,28^. These immune cells, as anti-cancer immune cells, release cytokines such as interferon-gamma (IFN-γ) and tumor necrosis factor (TNF)-alpha which are major effectors upregulating HLA class I molecules^29,30^. Thus, in HR-negative group, high levels of HLA class I expression in IBC compared to DCIS might be associated with increased immune cell infiltration caused by accumulation of genetic alterations during invasive tumor progression.

Considering the role of HLA class I in antigen presentation^31^, high expression of HLA class I would increase cancer cell recognition, and thus, result in increased CD8+ T cell infiltration. Several studies have demonstrated that HLA class I expression is associated with increased TILs. Na et al.^19^ reported that the density of CD3+ and CD8+ T cell infiltration was significantly correlated with higher expression of HLA class I in colorectal cancer. While Lee and colleagues^26^ reported that expression level of HLA class I related to higher TIL infiltration, especially CD8+ T cells in TNBC, Park et al.^23^ showed that increased HLA class I expression was associated with a higher number of Tregs in breast cancer. Our study revealed that a high level of HLA class I was associated with increased immune cell infiltration of not only CD8+ T cells but also immune-suppressive FOXP3+ Tregs and PD-L1+ immune cells in both DCIS and IBC. This finding also supports the possibility of induction of HLA class I expression by infiltrating immune cells rather than increased immune cell infiltration caused by increased HLA class I expression in tumor cells.

In this study, HLA class I expression showed a statistically significant impact on patient survival in HR-negative IBC, especially in TNBC. Although the connection between HLA class I down-regulation and patient prognosis is unclear, studies on the evolution of HLA class I expression during cancer progression may help explain this finding^32,33^. Genetic diversity caused by the accumulation of mutations may lead to the selection of the subclones that survive anti-cancer immunity. Cancer cells with alteration of HLA class I may be selected and they could escape T-cell-mediated immune destruction, leading to cancer progression. HR-negative breast cancers, especially TNBCs, are more immunogenic than the other subtypes, and thus, HLA class I down-regulation in TNBC might have a greater impact on immune surveillance resulting in poor clinical outcome of patients. However, there were conflicting results regarding the prognostic impact of HLA class I expression. Shin et al. reported that HLA class I heavy chain expression was associated with poor disease-free survival in patients with HR-positive breast cancer^17^. Kruijf and colleagues reported that HLA class I was not associated with clinical outcome in patients with breast cancer^34^. Nevertheless, loss of HLA class I has generally been reported to be related to worse survival in various cancers including breast cancer, non-small cell lung cancer, and colorectal cancer^19,23,35^. These contradictory results might be partially due to the use of different antibodies recognizing different HLA loci, various cutoffs for positive or negative staining, and different compositions of study populations. Further studies would be needed to validate the prognostic effect of HLA class I down-regulation in breast cancer.

In conclusion, our study showed that high expression of HLA class I expression was associated with aggressive clinicopathologic features of IBC. In addition, high HLA class I expression appeared to be related to increased immune cell infiltration including CD8+ T cells, CD4+ T cells, Treg and PD-L1+ immune cells. HLA class I was lost, but paradoxically its expression increased in association with increased immune cell infiltration during in situ to invasive progression of HR-negative breast cancers. Prognostic significance of HLA class I down-regulation was dependent on HR status, and it was found to affect patient prognosis in HR-negative breast cancers, especially in TNBCs.

## Materials and methods

### Tissue samples

A total of 830 breast cancer cases comprising 288 cases of DCIS (including 211 pure DCIS and 77 DCIS with microinvasion) and 542 cases of IBC that had been diagnosed between 2003 and 2011 at Seoul National University Bundang Hospital were included in this study. Of the 542 IBC cases, 90 cases with a sufficient DCIS component were selected for comparative analysis of the invasive and DCIS components within the same tumor. Patient details and clinicopathologic information were collected from the medical records and reviewing hematoxylin and eosin (H&E)-stained slides and immunohistochemical slides for the standard biomarkers. For DCIS, the following information was collected: age at diagnosis, tumor extent, nuclear grade, presence of comedo-type necrosis, microinvasion, the status of estrogen receptor (ER), progesterone receptor (PR), human epidermal growth factor receptor 2 (HER2), Ki-67 proliferation index, P53 overexpression, treatment modality, and clinical outcome. For IBC, clinicopathologic data including age at diagnosis, pT stage, pN stage, histologic subtype, histologic grade, biomarker status, adjuvant therapy, recurrence, and follow-up information were collected. The clinicopathological features of DCIS and IBC are summarized in Supplementary Tables S4 and S5, respectively. This study was approved by the institutional review board (IRB) of Seoul National University Bundang Hospital (IRB No B-1803/450–305), and informed consent was waived by the IRB. All experiments and procedures performed in studies involving human participants were in accordance with the ethical standards of the institutional research committee and with the 1964 Helsinki declaration and its later amendments or comparable ethical standards.

### Tissue microarrays (TMAs)

The most representative areas on H&E stained slides were selected, and the corresponding areas were taken from the formalin-fixed, paraffin-embedded tissue blocks in each case for tissue microarray (TMA) construction. One to three cores of 4-mm-diameter depending on the extent of the tumor from DCIS and three cores of 2-mm-diameter from IBC were arranged in TMA blocks using a trephine apparatus (Superbiochips Laboratories, Seoul, Korea).

### Evaluation of HLA class I expression by immunohistochemistry and scoring

Immunohistochemical staining of HLA class I was performed on TMAs using HLA-ABC (Clone EMR8-5; 1:200 dilution; Abcam) with Dako EnVision detection kit (Dako, Carpinteria, CA, USA). Some of the IBC cases had been evaluated for HLA-ABC expression in the previous study^36^, and those cases were re-evaluated during the study. HLA-ABC expression was evaluated in the tumor cell membrane for both intensity and proportion of positive cells. We adopted the interpretation method of HLA class I expression from the study by Na et al.^19^. The staining intensity was classified into negative, weak positive, and strong positive. Lymphocytes and endothelial cells were used as internal control, and weak positivity was defined as staining weaker than internal control. Staining equivalent or stronger than internal control was considered strong positive. For statistical analysis, high expression was defined as 50% or more tumor cells showing strong positivity. Low expression was defined as < 50% of the tumor cells revealing strong positivity or ≥ 50% showing weak positivity.

### Data of immune cell subsets

The data for the CD4+, CD8+, and FOXP3+ TILs and PD-L1+ immune cells were adopted from our previous studies^11,36^ for 288 cases of DCIS and 339 cases of IBC. Immunohistochemical staining had been performed with a BenchMark XT autostainer (Ventana Medical Systems) using an UltraView detection kit (Ventana Medical Systems). The following antibodies were used: CD4 (clone SP35; ready to use; Dako), CD8 (clone C8/144B; ready to use; Dako), FOXP3 (clone 236A/E7; 1:100; Abcam) and PD-L1 (clone E1L3N; 1:100; Cell Signaling, Danvers, MA, USA).

CD4+, CD8+, and FOXP3+ T cells had been counted in intratumoral and stromal areas as absolute numbers per high-power field. Detailed information on the counting method of TILs is described in the previous studies^11,36^. PD-L1+ immune cells were considered to be present when at least 1% of the tumor stromal area was occupied by PD-L1+ immune cells.

### Evaluation of standard biomarkers and determination of breast cancer subtypes

Expression of the basic biomarkers including ER, PR, HER2, p53, and Ki-67 was evaluated at the time of pathologic diagnosis in the surgical specimens. The antibodies stained were as follows; ER (clone SP1; 1:100 dilution; LabVision, Fremont, CA), PR (clone PgR 636; 1:70 dilution; Dako, Carpinteria, CA), HER2 (clone 4B5; ready to use; Ventana Medical Systems, Tuscon, AZ), p53 (clone D07; 1:600 dilution; Dako), and Ki-67 (clone MIB-1; 1:250 dilution; Dako). ER and PR staining in ≥ 1% of the tumor cells was determined as positive. Patients with ER- or PR-positive tumors were regarded as hormonal receptor (HR)-positive. HER2 positivity was defined as an immunohistochemical score of 3+ or the presence of HER2 gene amplification on fluorescence/silver in situ hybridization. For p53, staining in 10% or more of the tumor cells was considered positive. High Ki-67 proliferation index was defined as staining in ≥ 10% of tumor cells for DCIS and ≥ 20% for IBC.

Breast cancer subtype was determined with standard biomarker profiles according to 2011 St Gallen International Expert Consensus^37^. Each subtype was defined as follows: luminal A (ER+ and/or PR+, HER2−, Ki-67 < 14%), luminal B (ER+ and/or PR+, HER2−, Ki-67 ≥ 14%; ER+ and/or PR+, HER2+), HER2+ (ER−, PR−, HER2+), and triple-negative (ER−, PR−, HER2−).

### Statistical analysis

All statistical analysis was performed using Statistical package, SPSS version 22.0 for Windows (SPSS Inc, Chicago, IL). The Chi-square test or Fisher’s exact test was used to analyze the association between HLA class I expression and clinicopathologic characteristics of DCIS and IBC or status of PD-L1+ immune cells. Comparison of HLA class I expression between the invasive and in situ components within the same tumor was performed using McNemar test. Difference in CD4+, CD8+, and FOXP3+ TILs was analyzed by the Mann–Whitney U test between the two groups. Recurrence-free survival for DCIS and disease-free survival for IBC were assessed by the Kaplan–Meier method with the log-rank test. Factors that affected survival were identified using univariate analysis, which were then used for multivariate analysis using a backward stepwise selection method in the Cox proportional hazard model. Statistical significance was based on *p* < 0.05 with all reported *p* values being two-sided.

## Supplementary Information

Supplementary Tables.

Supplementary Figure 1.

Supplementary Legends.

## Data Availability

The datasets used and/or analyzed during the current study are available from the corresponding author on reasonable request.
