# Supplementary material for: Expression of HLA class I is associated with immune cell infiltration and patient outcome in breast cancer
Source: Sci Rep. 2022 Nov 27;12:20367. doi: 10.1038/s41598-022-24890-3 (PMC9701770; doi:10.1038/s41598-022-24890-3)
Supplement: Supplementary file 1 — Supplementary Tables. [file 41598_2022_24890_MOESM1_ESM.docx]

**Supplementary Table S1. Comparison of HLA class I expression between DCIS and DCIS associated with invasive carcinoma (DCIS-INV)**

| **Group** | **HLA class I expression** | **DCIS**  **(n=288)** | **DCIS-INV**  **(n=90)** | ***p value*** |
| --- | --- | --- | --- | --- |
| Total | Low | 145 (50.3%) | 44 (48.9%) | 0.809 |
|  | High | 143 (49.7%) | 46 (51.1%) |  |
|  | Complete loss | 27 (9.4%) | 11 (12.2%) | 0.433 |
| HR-positive | Low | 106 (46.9%) | 35 (52.2%) | 0.443 |
|  | High | 120 (53.1%) | 32 (47.8%) |  |
|  | Complete loss | 22 (9.7%) | 8 (11.9%) | 0.601 |
| HR-negative | Low | 39 (62.9%) | 9 (39.1%) | 0.050 |
|  | High | 23 (37.1%) | 14 (60.9%) |  |
|  | Complete loss | 5 (8.1%) | 3 (13.0%) | 0.677 |

*P* values are calculated by Chi-square test.

DCIS; ductal carcinoma in situ; HR, hormone receptor

**Supplementary Table S2. Relationship between complete loss of HLA class I and clinicopathologic features of DCIS**

| **Clinicopathologic feature** | **HLA class I** | | ***p* value** |
| --- | --- | --- | --- |
|  | **Complete loss (n=27)** | **Any expression (n=261)** |  |
| Extent (cm) |  |  | 0.078 |
| <3.0 | 17 (63.0) | 118 (45.2) |  |
| ≥3.0 | 10 (37.0) | 143 (54.8) |  |
| Nuclear grade |  |  | 0.502 |
| Low to intermediate | 16 (59.3) | 137 (52.5) |  |
| High | 11 (40.7) | 124 (47.5) |  |
| Comedo-type necrosis |  |  | 0.435 |
| Absent | 20 (74.1) | 174 (66.7) |  |
| Present | 7 (25.9) | 87 (33.3) |  |
| Microinvasion |  |  | 0.005 |
| Absent | 26 (96.3) | 185 (70.9) |  |
| Present | 1 (3.7) | 76 (29.1) |  |
| Estrogen receptor |  |  | 0.597 |
| Negative | 5 (18.5) | 60 (23.0) |  |
| Positive | 22 (81.5) | 201 (77.0) |  |
| Progesterone receptor |  |  | 0.427 |
| Negative | 6 (22.2) | 77 (29.5) |  |
| Positive | 21 (77.8) | 184 (70.5) |  |
| HER2 status |  |  | 0.540 |
| Negative | 22 (81.5) | 199 (76.2) |  |
| Positive | 5 (18.5) | 62 (23.8) |  |
| Ki67 index |  |  | 0.812 |
| Low (<10%) | 18 (66.7) | 168 (64.4) |  |
| High (≥10%) | 9 (33.3) | 93 (35.6) |  |
| P53 overexpression |  |  |  |
| Absent | 25 (92.6) | 209 (80.1) |  |
| Present | 2 (7.4) | 52 (19.9) |  |
| Subtype |  |  | 0.735 |
| Luminal A | 18 (66.7) | 163 (62.5) |  |
| Luminal B | 4 (14.8) | 41 (15.7) |  |
| HER2+ | 2 (7.4) | 37 (14.2) |  |
| Triple negative | 3 (11.1) | 20 (7.7) |  |

HLA, human leukocyte antigen; DCIS, ductal carcinoma in situ

**Supplementary Table S3. Relationship between complete loss of HLA class I and clinicopathologic features of IBC**

| **Clinicopathologic features** | **HLA class I** | | | ***p* value** |
| --- | --- | --- | --- | --- |
|  | **Complete loss (n=92)** | **Any expression (n=450)** | |  |
| T stage |  | |  | 0.306 |
| T1 | 40 (43.5) | | 222 (49.3) |  |
| T2-T4 | 52 (56.5) | | 228 (50.7) |  |
| N stage |  | |  | 0.323 |
| N0 | 48 (52.2) | | 260 (578) |  |
| N1-N3 | 44 (47.8) | | 190 (42.2) |  |
| Histologic grade |  | |  | 0.108 |
| I-II | 52 (56.5) | | 213 (47.3) |  |
| III | 40 (43.5) | | 237 (52.7) |  |
| Lymphovascular invasion |  | |  | 0.948 |
| Absent | 52 (56.5) | | 256 (56.9) |  |
| Present | 40 (43.5) | | 194 (43.1) |  |
| Estrogen receptor |  | |  | 0.281 |
| Negative | 24 (26.1) | | 143 (31.8) |  |
| Positive | 68 (73.9) | | 307 (68.2) |  |
| Progesterone receptor |  | |  | 0.843 |
| Negative | 37 (40.2) | | 176 (39.1) |  |
| Positive | 55 (59.8) | | 274 (60.9) |  |
| HER2 status |  | |  | 0.084 |
| Negative | 77 (83.7) | | 339 (75.3) |  |
| Positive | 15 (16.3) | | 111 (24.7) |  |
| Ki67 index |  | |  | 0.033 |
| Low (<20%) | 61 (66.3) | | 244 (54.2) |  |
| High (≥20%) | 31 (33.7) | | 206 (45.8) |  |
| P53 overexpression |  | |  | 0.020 |
| Absent | 77 (83.7) | | 324 (72.0) |  |
| Present | 15 (16.3) | | 126 (28.0) |  |
| Subtype |  | |  | 0.055 |
| Luminal A | 50 (54.3) | | 182 (40.4) |  |
| Luminal B | 18 (1936) | | 134 (29.8) |  |
| HER2+ | 12 (13.0) | | 52 (11.6) |  |
| Triple negative | 12 (13.0) | | 82 (18.2) |  |

HLA, human leukocyte antigen; IBC, invasive breast carcinoma

**Supplementary Table S4. Clinicopathologic characteristics of DCIS**

| **Characteristic** | **Number (%)** |
| --- | --- |
| Age at diagnosis, years |  |
| Median (range) | 48 (26-88) |
| Extent, cm |  |
| Median (range) | 3.0 (0.4-14.5) |
| Nuclear grade |  |
| Low | 16 (5.6) |
| Intermediate | 137 (47.6) |
| High | 135 (46.9) |
| Comedo-type necrosis |  |
| Absent | 74 (25.7) |
| Present | 214 (74.3) |
| Microinvasion |  |
| Absent | 211 (73.3) |
| Present | 77 (26.7) |
| Estrogen receptor |  |
| Negative | 65 (22.6) |
| Positive | 223 (77.4) |
| Progesterone receptor |  |
| Negative | 83 (28.8) |
| Positive | 205 (71.2) |
| Hormone receptor |  |
| Negative | 62 (21.5) |
| Positive | 226 (28.5) |
| HER2 status |  |
| Negative | 221 (76.7) |
| Positive | 67 (23.3) |
| Ki67 index |  |
| Low (<10%) | 186 (64.6) |
| High (≥10%) | 102 (35.4) |
| P53 overexpression |  |
| Absent | 234 (81.3) |
| Present | 54 (18.8) |
| Subtype |  |
| Luminal A | 181 (62.8) |
| Luminal B | 45 (15.6) |
| HER2+ | 39 (13.5) |
| Triple negative | 23 (8.0) |
| Adjuvant radiation therapy |  |
| Not received | 130 (45.1) |
| Received | 158 (54.9) |
| Adjuvant hormonal therapy |  |
| Not received | 181 (62.8) |
| Received | 107 (37.2) |

DICS, ductal carcinoma in situ

**Supplementary Table S5. Clinicopathologic characteristics of IBC**

| **Characteristic** | **Number (%)** |
| --- | --- |
| Age at diagnosis, years |  |
| Median (range) | 49 (20-87) |
| T stage |  |
| T1 | 262 (48.3) |
| T2 | 255 (47.0) |
| T3 | 17 (3.1) |
| T4 | 4 (1.5) |
| N stage |  |
| N0 | 308 (56.8) |
| N1 | 144 (26.6) |
| N2 | 50 (9.2) |
| N3 | 40 (7.4) |
| Histologic subtype |  |
| Invasive carcinoma of no special type | 479 (88.38) |
| Mucinous carcinoma | 16 (2.95) |
| Metaplastic carcinoma | 13 (2.40) |
| Others | 34 (6.27) |
| Histologic grade |  |
| I | 100 (18.5) |
| II | 165 (30.4) |
| III | 277 (51.1) |
| Lymphovascular invasion |  |
| Absent | 308 (56.8) |
| Present | 234 (43.2) |
| Estrogen receptor |  |
| Negative | 167 (30.8) |
| Positive | 375 (69.2) |
| Progesterone receptor |  |
| Negative | 213 (39.3) |
| Positive | 329 (60.7) |
| Hormone receptor |  |
| Negative | 158 (29.2) |
| Positive | 384 (70.8) |
| HER2 status |  |
| Negative | 416 (76.8) |
| Positive | 126 (23.2) |
| Ki67 index |  |
| Low (<20%) | 305 (56.3) |
| High (≥20%) | 237 (43.7) |
| P53 overexpression |  |
| Absent | 401 (74.0) |
| Present | 141 (26.0) |
| Subtype |  |
| Luminal A | 233 (42.8) |
| Luminal B | 152 (28.0) |
| HER2+ | 64 (11.8) |
| Triple negative | 94 (17.3) |
| Adjuvant chemotherapy^*^ |  |
| Not received | 93 (17.3) |
| Received | 443 (82.7) |
| Adjuvant HER2-targeted therapy^*^ |  |
| Not received | 475 (88.62) |
| Received | 61 (11.38) |
| Adjuvant radiation therapy^*^ |  |
| Not received | 225 (41.98) |
| Received | 311 (58.02) |
| Adjuvant hormonal therapy^*^ |  |
| Not received | 166 (30.98) |
| Received | 370 (69.02) |

*****Information was available for 536 patients.

IBC, invasive breast carcinoma
