# Supplementary material for: Expression of HLA class I is associated with immune cell infiltration and patient outcome in breast cancer
Source: Sci Rep. 2022 Nov 27;12:20367. doi: 10.1038/s41598-022-24890-3 (PMC9701770; doi:10.1038/s41598-022-24890-3)
Supplement: Supplementary file 3 — Supplementary Legends. [file 41598_2022_24890_MOESM3_ESM.docx]

**Supplementary Figure 1**. Kaplan–Meier survival curves according to the expression level of human leukocyte antigen (HLA) class I in patients with ductal carcinoma in situ (DCIS). Expression level of HLA class I is not associated with recurrence-free survival of patients with DCIS, irrespective of hormone receptor status.
